# Improved Exercise Tolerance with Caffeine Is Associated with Modulation of both Peripheral and Central Neural Processes in Human Participants

**DOI:** 10.3389/fnut.2018.00006

**Published:** 2018-02-12

**Authors:** Joanna L. Bowtell, Magni Mohr, Jonathan Fulford, Sarah R. Jackman, Georgios Ermidis, Peter Krustrup, Katya N. Mileva

**Affiliations:** ^1^Sport and Health Sciences, College of Life and Environmental Sciences, Exeter University, Exeter, United Kingdom; ^2^Centre of Health Science, Faculty of Health Sciences, University of the Faroe Islands, Tórshavn, Faroe Islands; ^3^Centre of Health and Human Performance, Department of Food and Nutrition, and Sport Science, University of Gothenburg, Gothenburg, Sweden; ^4^Exeter NIHR Clinical Research Facility, Medical School, University of Exeter, Exeter, United Kingdom; ^5^Department of Exercise and Wellness, Parthenope University of Naples, Naples, Italy; ^6^Department of Sports Science and Clinical Biomechanics, Sport and Health Sciences Cluster (SHSC), University of Southern Denmark, Odense, Denmark; ^7^Sport and Exercise Science Research Centre, School of Applied Science, London South Bank University, London, United Kingdom

**Keywords:** caffeine, fatigue, transcranial magnetic stimulation, neuromuscular function, central fatigue

## Abstract

**Background:**

Caffeine has been shown to enhance exercise performance and capacity. The mechanisms remain unclear but are suggested to relate to adenosine receptor antagonism, resulting in increased central motor drive, reduced perception of effort, and altered peripheral processes such as enhanced calcium handling and extracellular potassium regulation. Our aims were to investigate how caffeine (i) affects knee extensor PCr kinetics and pH during repeated sets of single-leg knee extensor exercise to task failure and (ii) modulates the interplay between central and peripheral neural processes. We hypothesized that the caffeine-induced extension of exercise capacity during repeated sets of exercise would occur despite greater disturbance of the muscle milieu due to enhanced peripheral and corticospinal excitatory output, central motor drive, and muscle contractility.

**Methods:**

Nine healthy active young men performed five sets of intense single-leg knee extensor exercise to task failure on four separate occasions: for two visits (6 mg·kg^−1^ caffeine vs placebo), quadriceps ^31^P-magnetic resonance spectroscopy scans were performed to quantify phosphocreatine kinetics and pH, and for the remaining two visits (6 mg·kg^−1^ caffeine vs placebo), femoral nerve electrical and transcranial magnetic stimulation of the quadriceps cortical motor area were applied pre- and post exercise.

**Results:**

The total exercise time was 17.9 ± 6.0% longer in the caffeine (1,225 ± 86 s) than in the placebo trial (1,049 ± 73 s, *p* = 0.016), and muscle phosphocreatine concentration and pH (*p* < 0.05) were significantly lower in the latter sets of exercise after caffeine ingestion. Voluntary activation (VA) (peripheral, *p* = 0.007; but not supraspinal, *p* = 0.074), motor-evoked potential (MEP) amplitude (*p* = 0.007), and contractility (contraction time, *p* = 0.009; and relaxation rate, *p* = 0.003) were significantly higher after caffeine consumption, but at task failure MEP amplitude and VA were not different from placebo. Caffeine prevented the reduction in M-wave amplitude that occurred at task failure (*p* = 0.039).

**Conclusion:**

Caffeine supplementation improved high-intensity exercise tolerance despite greater-end exercise knee extensor phosphocreatine depletion and H^+^ accumulation. Caffeine-induced increases in central motor drive and corticospinal excitability were attenuated at task failure. This may have been induced by the afferent feedback of the greater disturbance of the muscle milieu, resulting in a stronger inhibitory input to the spinal and supraspinal motor neurons. However, causality needs to be established through further experiments.

## Introduction

Caffeine (1, 2, 3 methylxanthine) supplementation has been demonstrated to improve endurance (1), high-intensity (2) and strength exercise capacity, and performance (3) at doses ranging from 2 to 10 mg·kg^−1^. Caffeine is rapidly absorbed into the bloodstream after ingestion, with peak concentrations [~70 μM after 6 mg·kg^−1^ (4)] achieved 30–60 min after ingestion. The mechanisms of action remain unclear but have been suggested to include an increased central motor drive and a reduced perception of effort related to adenosine receptor antagonism (5), as well as an increased muscle contractility due to altered calcium kinetics and/or sensitivity and an improved muscle ion regulation (6, 7).

Adenosine has inhibitory effects on the central nervous system excitability by attenuating the release of excitatory neurotransmitters and reducing neuronal-firing rates (8). Caffeine has a similar structure to adenosine and as a consequence is an effective adenosine receptor antagonist (9). Caffeine has been shown to increase excitatory neurotransmitter release and lower the activation threshold of cerebral cortical neurons (10), as well as increase serotonin levels in the serotonergic neurons of the raphe nuclei in the brainstem of the rat (11). This may cause an increased spinal motor neuron excitability since there is an excitatory input from the raphe nuclei at the spinal level (12). There is also evidence that this translates to human *in vivo* physiology, since there is an increased incidence of self-sustained firing of tibialis anterior motor units after caffeine supplementation (13).

The effects of caffeine on corticospinal excitability have been assessed in a small number of studies using transcranial magnetic stimulation (TMS). The motor-evoked potential (MEP) amplitude was elevated in the vastus lateralis muscle sustaining a contraction at 3% MVC, 1 h after consuming 6 mg·kg^−1^ caffeine, and the fatigue-induced decline in MEP amplitude was attenuated by caffeine (14). The extension of time-to-task failure in this open-ended single-leg knee extensor task after caffeine ingestion was correlated with MEP amplitude at the start of exercise, clearly implicating an increased corticospinal excitability as an important mechanism of caffeine’s action. Caffeine has also consistently been shown to increase central drive, measured as knee extensor voluntary activation (VA), in both fresh (14, 15) and fatigued (15) muscle, and a meta-analysis (1) indicates a moderate- to large-effect size of caffeine on VA (0.67 effect size).

Peripheral mechanisms are also involved in caffeine’s actions. *In vitro* studies have demonstrated improved excitation–contraction coupling *via* increased twitch and tetanic force produced during electrically evoked contractions. The mechanisms proposed include the antagonism of adenosine A_1_ receptors on the muscle membrane, as well as an interaction with the ryanodine receptors of the sarcoplasmic reticulum (SR), thus increasing calcium release and myofibrillar calcium sensitivity and reducing SR Ca^2+^-ATPase sensitivity [for a review, see Ref. (16)]. However, these studies employed supraphysiological doses of caffeine (10 mM). More recently, both James et al. (17) and Tallis et al. (18) demonstrated enhanced twitch torque in isolated mouse muscle at physiologically relevant concentrations (50–70 μM). In addition, some human studies have shown increases in resting twitch torque amplitude and temporal characteristics in fresh and fatigued muscle after caffeine supplementation, suggesting altered calcium handling. Extracellular potassium accumulation is implicated in fatigue development since this will result in the depolarization of the resting membrane, resulting in a broadened action potential, a reduced conduction velocity, and a decreased excitability (19). Shushakov et al. (20) observed an inverse relationship between M-wave area and venous potassium concentration during fatiguing static and dynamic exercise. The accumulation of extracellular potassium normally observed during fatiguing high-intensity exercise seems to be attenuated after caffeine ingestion. Caffeine ingestion (6 mg·kg^−1^) attenuated the increase in interstitial potassium during one-legged knee extensor exercise at 20 (10 min) and 50 W (3 × 3 min) measured using microdialysis (21). In the same study, high-intensity capacity was improved by 16% after caffeine ingestion. Possible mechanisms are suggested to include elevated catecholamine concentrations that result in increased Na^+^–K^+^ ATPase activity, thus supporting the maintenance of the resting membrane potential and presumably therefore better preserved membrane excitability.

Caffeine affects peripheral and central neural processes and therefore provides an excellent tool to study the integration of afferent sensory information within regions upstream of the motor cortex to produce an appropriate level of central motor drive to preserve function during fatiguing exercise. There is a lack of data on the effects of caffeine on muscle metabolism, and certainly no studies have been conducted where both muscle metabolism and neuromuscular function have been assessed after caffeine ingestion. We employed magnetic resonance spectroscopy (MRS) of the quadriceps to allow continuous quantification of muscle metabolites and pH changes during exercise. In a parallel set of experiments, TMS and electrical stimulation of the femoral nerve were utilized to assess central and peripheral neural processes. The aim of the present study was to determine whether the caffeine-induced extension of time-to-task failure during repeated sets of high-intensity single-leg knee extensor exercise was related to altered interplay between central and peripheral neural processes and despite greater changes in the knee extensor metabolites. We hypothesized that caffeine ingestion will extend the time-to-task failure *via* augmented peripheral and corticospinal excitatory output and central motor drive despite greater declines in knee extensor phosphocreatine concentration and pH at task failure.

## Materials and Methods

### Participants

Nine male recreational athletes [age: 26.0 ± 2.7 (±SEM) years; height: 1.79 ± 0.02 m; weight: 78.4 ± 2.2 kg] took part in this double-blind part-randomized controlled study, which was approved by the Sport and Health Sciences Research Ethics Committee at the Exeter University and conducted in accordance with the Declaration of Helsinki. After being informed verbally and in writing of the experimental procedures and associated risks, all participants completed a medical health questionnaire and a screening questionnaire to ensure that there were no contraindications for magnetic resonance scanning and cortical stimulation, before providing written informed consent to the experimental procedures. All participants were non-smokers, habitual caffeine consumers, in good health, and had no known history of cardiovascular, metabolic, neurological, or motor disorder. On test days, participants were instructed to report to the laboratory in a rested and fasted state, having completed no strenuous exercise or consumed alcohol or caffeine within the previous 24 h.

### Experimental Design

After a familiarization visit, each participant completed four main trials in the morning, separated by between 3 and 7 days. In each main trial, they performed five sets of intense single-leg knee extensor exercise to task failure, separated by a 5-min rest. On the first two visits, ^31^P-MRS measurements of the quadriceps were made throughout exercise, and for the remaining two visits, the peripheral and corticospinal excitability and the contractility of the quadriceps muscle were evaluated using peripheral nerve electrical (PNS) and transcranial magnetic (TMS) stimulation. Trial order for both MRI and the neuromuscular function measurement visits, placebo (glucose) or caffeine (6 mg·kg^−1^) was randomized using a sealed envelope system.

### Protocol

On arrival at the laboratory for the first two visits, a cannula was inserted into the antecubital vein, and venous blood samples were collected at regular intervals throughout the protocol. In order to collect ^31^P data, a 6-cm ^31^P transmit/receive surface coil was placed within the bed of a 1.5-T whole-body MR scanner (Intera, Philips, The Netherlands) at the Exeter Magnetic Resonance Research Centre (Exeter, UK). The participants were positioned in a prone position such that the coil was centered over the quadriceps muscle of the right leg. After images were acquired to determine the correct positioning of the muscle relative to the coil, pre-acquisition steps were carried out to optimize the signal from the muscle under investigation. This comprised the tuning and matching of the coil to maximize energy transfer between the coil and the muscle, and an automatic shimming protocol to optimize the homogeneity of the local magnetic field within a volume that defined the quadriceps muscle.

Initial resting baseline scans for the determination of metabolite concentrations were obtained with a repetition time of 20 s in order to avoid saturation effects and were averaged over 36 individual spectra. Participants were then removed from the scanner and provided with either caffeine or placebo supplement. After 1 h, they were returned to the scanner and set-up procedures as described for the baseline measurements repeated. Following 1-min of rest, participants undertook one-legged knee extension exercise which resulted in the lifting and lowering of weights *via* a pulley system, a well-established exercise model in our laboratories (22). In all cases, the weight to be lifted was participant-specific (8.7 ± 0.6 kg) and had been determined in an incremental test performed several days earlier, in which the weight resulting in task failure within approximately 5 min was selected. The extensions took place at a frequency of 0.67 Hz with the foot moving over a distance of ~0.22 m (each complete cycle lasted 1.5 s), with audio cues being presented to ensure that the rate of contraction was maintained at the desired value. Exercise was continued until task failure, as defined by the inability to maintain exercise with the specified rate and range of movement. Following a 5-min recovery, another exercise bout to task failure was undertaken with the process repeated until five sets had been completed. During the entire exercise protocol (including the recovery periods), ^31^P data were acquired every 1.5 s and phase cycling with four phase cycles employed, leading to a spectra being acquired every 6.0 s.

For the final two visits, participants returned to the laboratory, at the same time of the day as for the first trial. A surface EMG electrode was positioned over *musculus vastus medialis* (VM) and *musculus biceps femoris* (BF) of the right leg, to record voluntary EMG activity and evoked potentials. Next, the adhesive anode and cathodes were attached as described below for femoral nerve stimulation. Participants were then positioned in a mock MRI construction in a prone position in order to replicate the position during MRS measurements. In order to measure maximum knee extensor force (MVC) during isometric contractions while in the prone position adopted in this study, the right leg was flexed and the shin placed against a solid plastic block attached to a calibrated force transducer, all of which was fixated to the plinth. Participants were firmly strapped to the plinth at the shoulders, waist, hips, and mid-thigh to prevent extraneous movement. Isometric force produced by the knee extensors was recorded as participants pushed the shin toward the plinth. After identification of the optimal stimulation points for obtaining maximal responses in VM to PNS and TMS (see the procedure below), the VM M-wave recruitment curve was constructed to determine the maximal M-wave (Mmax). The active motor threshold (aMT) for TMS was then established as described below.

**Figure 1:**
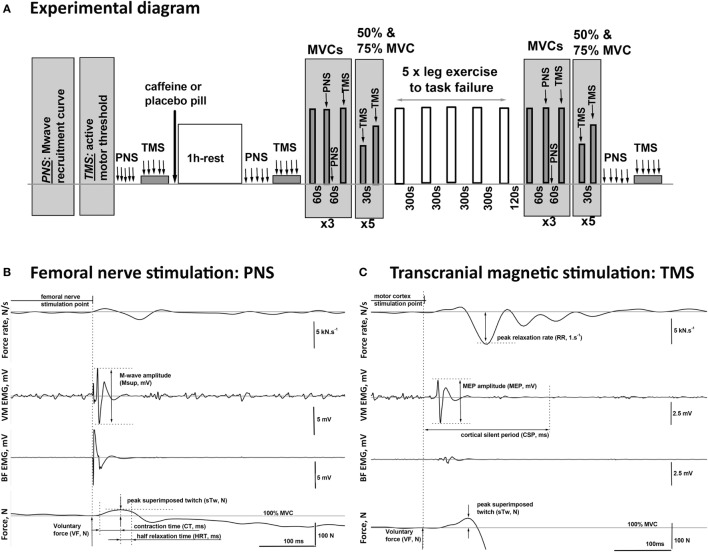
Schematic diagram of the experimental procedures. **(A)** One hour after consuming either caffeine (6 mg·kg^−1^) or placebo supplement, the participants completed five sets of one-legged knee extension exercise (lifting and lowering of weights *via* a pulley system in a prone position) to task failure in two trials, each consisting of two visits. On the visits in the second trial, peripheral nerve (PNS) and transcranial magnetic (TMS) stimulation protocols were performed before and 1 h after the supplement intake, as well as after the completion of the exercise. **(B,C)** Examples of the EMG and twitch responses to stimulations of the PNS and the motor cortex (TMS) affiliated with the muscles in the upper leg recorded during maximal (100% MVC) and submaximal (75% MVC) voluntary contractions, respectively. The illustrated responses were recorded from the agonist [vastus medialis (VM)] and the antagonist [biceps femoris (BF)] muscles of the exercised leg of a representative participant. The diagram illustrates the method for the calculation of the characteristic time–amplitude parameters of the evoked responses.

Participants received five single supramaximal (130% Mmax) PNS pulses in the resting state and five single TMS pulses at 120% aMT while maintaining a 5% MVC contraction before consuming a pill (placebo or caffeine). PNS and TMS were repeated 60 min after consumption of the pill, followed by nine MVCs each lasting about 3 s and separated by a 1-min rest (Figure 1A). The first MVC was non-stimulated, then during and 2 s after the second MVC supramaximal PNS was delivered, and during the third MVC supramaximal TMS was delivered. This sequence was then repeated twice more. Participants were then asked to perform 10 brief submaximal contractions at either 50 or 75% MVC with visual feedback of the target level and the force production provided. Single suprathreshold TMS pulses were delivered during each of these contractions. Participants then completed the five sets of one-legged knee extension exercise as described above. The PNS and TMS protocols were then repeated, commencing 2 min after task failure and lasting for ~15 min, except that the resting femoral nerve stimulations and TMS during 5% MVC contractions were performed at the end of the test battery on this occasion (Figure 1A).

### Peripheral Nerve Electrical Stimulation

Supramaximal single-pulse electrical stimuli (200-μs pulse duration) were delivered to the right femoral nerve with a high-voltage direct current stimulator (Digitimer DS7AH, Digitimer Ltd., Welwyn Garden City, UK). The cathode was placed in the femoral triangle, 2 cm lateral to the femoral artery, and an adhesive anode (both 2 cm × 2 cm biotab adhesive electrodes, Boots UK Ltd., Nottingham, England) placed in the gluteal fold opposite the cathode. The cathode was systematically moved vertically and horizontally, and the amplitude of the compound muscle action potential (CMAP, M-wave) was monitored to identify the optimal position of the cathode for attaining maximal peak-to-peak M-wave amplitude. The stimulation intensity, which elicited Mmax, was identified by increasing the femoral nerve PNS stimulation current intensity in 10-mA increments from 100 mA. All subsequent stimulations were delivered at 130% of the Mmax intensity to ensure supramaximal stimulations (223 ± 11 mA).

### Transcranial Magnetic Stimulation

Motor-evoked potentials were elicited in the knee extensor muscles by TMS of the contralateral motor cortical leg area (Magstim 200 stimulator, Magstim Company Ltd., UK). Single pulses of up to 2-T intensity (100% Magstim output) were delivered to the motor cortex through 110° double cone coil (9-cm diameter each, type P/N 9902-00). The optimal coil position was determined by moving the coil along a virtual grid lateral and posterior to the vertex (±3 cm) with a resolution of 1 cm until reaching a position at which maximal MEP amplitude in the slightly pre-activated VM (5% MVC) was produced with minimal stimulation intensity. To optimize the stimulation intensity, the stimulus–response curve was constructed for each subject by systematically increasing the TMS intensity in 5% increments from 35% output followed by refinement steps of 1%. The aMT was defined as the lowest TMS intensity, eliciting an MEP in the VM muscle of minimum 200-μV peak-to-peak amplitude in at least 50% of the stimulations during a knee extension contraction at 5% MVC (23). The stimulation intensity during the exercise protocols was then set to 120% aMT (67 ± 3% stimulator output).

### Data Collection

Voluntary EMG activity level, CMAPs (M-waves), and MEPs were recorded from VM muscle of the right leg using surface-active bipolar bar EMG electrodes (99.9% Ag, 10-mm length, 1-mm width, 10-mm pole spacing, CMRR >80 dB, model DE2.1, DelSys Inc., Boston, MA, USA). The electrode was placed over the muscle belly at the recommended site for surface EMG recording from VM muscle (24). The ground electrode was placed over the patella of the right leg. Double-sided adhesive interfaces and hypoallergenic medical tape were used to ensure the stability of the contact between the EMG sensor and the skin. The skin area underneath the EMG electrode was shaved, then exfoliated with abrasive gel, and cleaned with ethyl propanol to minimize the skin impedance. The EMG signal was preamplified (×1,000) and band-pass filtered between 20 and 450 Hz at the source (Bagnoli-8, DelSys Inc., Boston, MA, USA), and then transferred to a computer with a sampling frequency of 2 kHz and high-pass filtered at 10 Hz.

Isometric knee extension force produced during the TMS and PNS protocols was measured using an in-line force transducer (Model: SSM-HN-250, Interface Force Measurements, Crowthorne, England,) which worked under compression and recorded the forces applied as the participant pushed their lower leg down toward the bed. The force transducer was fitted within a bespoke assembly, which was fixed onto the mock MRI scanner bed. Throughout the protocol, force data were sampled with a frequency of 200 Hz and recorded continuously and synchronously with the EMG signals in a PC *via* an A/D converter (CED 1401power, Cambridge, UK), using Spike2 data acquisition software (CED, Cambridge, UK) with a 16-bit resolution.

### Data Analysis

Data recorded during PNS and TMS protocols (visits three and four) were analyzed off-line using custom-written scripts developed in Spike2 ver.7 software (CED, Cambridge, UK), and an exemplar record indicating the extracted parameters of the evoked EMG and twitch responses is provided for illustrative purposes (Figures 1B,C).

### Muscle Metabolites

For both baseline and exercise protocols, MRS data were quantified by peak fitting, with the assumption of prior knowledge, using the AMARES fitting algorithm in the jMRUI (version 3) software package. Spectra were fitted assuming the presence of the following peaks: Pi, phosphodiester, PCr, α-ATP (two peaks, amplitude ratio 1:1), γ-ATP (two peaks, amplitude ratio 1:1), and β-ATP (three peaks, amplitude ratio 1:2:1). Resting molar concentrations of PCr and Pi were subsequently calculated *via* the ratio of PCr:ATP and Pi:ATP from the baseline spectra, assuming a resting concentration of ATP of 8.2 mM (25). Intracellular pH was calculated using the chemical shift of the Pi spectra relative to the PCr peak (26).

### Blood Analysis

Venous blood samples were drawn into 5-mL heparinized syringes (Terumo Corporation, Leuven, Belgium) from a cannula (Insyte-W™, Becton-Dickinson, Madrid, Spain) inserted into the subject’s antecubital vein. The blood was analyzed for (lactate) and (glucose) within ~2 min of collection (YSI 2300, Yellow Springs Instruments, Yellow Springs, OH, USA), and pH, pCO_2_, and HCO_3_^−^ were measured using ABL90 Flex analyzer (Radiometer Medical, Bronshoj, Denmark).

### Exercise Performance

The time-to-task failure in each set of exercise for each visit was measured, and data were averaged across the two visits for each condition (placebo and caffeine). The maximum isometric knee extension force (MVC, N) was evaluated before and after the exercise protocol and calculated as the average value over a 1-s period around the plateau level of the highest torque achieved in the three non-stimulated MVCs completed at each measurement time point (Figure 1A). Voluntary force (VF, N) during the contractions in which PNS or TMS was applied was calculated as the mean force level achieved during the 50 ms preceding the stimulation pulse delivery (Figures 1B,C).

### Excitatory Output

The peak-to-peak amplitude and the total area of the maximal VM M-wave (Mmax) evoked by PNS were measured pre- and post pill ingestion as well as after the exercise protocol at rest, during, and 2 s after MVC to quantify the excitability of the peripheral circuitry (distal motor axon, neuromuscular junction, and muscle fiber membrane) (Figure 1B). The corticospinal excitability to VM was quantified at each stimulation point by the MEP peak-to-peak amplitude and the total area normalized to the Mmax amplitude and area, respectively, as acquired at the corresponding activation level and time point. The excitability of the intracortical inhibitory pathways was assessed by the duration of the silent period (CSP, ms) measured between the stimulation point and the time when the muscle activation returned to the pre-stimulation voluntary EMG level. This was assessed by calculating the RMS EMG amplitude in the 500 ms preceding the delivery of each TMS pulse (Figure 1C). In addition, BF EMG data were monitored throughout the protocol to ensure that stimulation did not evoke potentials within BF, demonstrating that the activation of antagonist motor areas did not occur (exemplar data shown in Figures 1B,C).

### Peripheral Contractility

The peripheral contractility during maximal voluntary effort was quantified by the maximal muscle relaxation rate (RR) which occurs during the TMS-evoked cortical silent period. RR was identified as the minimum of the time derivative of the force signal and normalized (1/s) to the overall force calculated as the sum of the VF and the evoked superimposed twitch (sTw + VF, N). The superimposed twitch (sTw, N) was calculated as the peak force increment evoked by TMS above the VF, N level (Figure 1B). Peripheral contractility during MVC was also assessed from the peak amplitude, the contraction (CT, ms), and half relaxation (HRT, ms) times of the twitch evoked by peripheral nerve stimulation (PNS) (Figure 1B).

Peripheral contractility was evaluated by the size of the potentiated twitch (potTw, N) evoked by PNS delivered immediately after MVC. In addition, the twitch responses to TMS during contractions at 50, 75, and 100% MVC were used to estimate the twitch evoked by TMS (eRTw, N).

### Voluntary Activation

Neural drive during the VA of the motoneuron pool (VA, %) was evaluated from the superimposed (sTw) and the potentiated (potTw) twitches evoked by PNS during and 2 s after the MVCs, respectively, using the standard twitch interpolation equation: VA = (1 − sTw/potTw) × 100%. Supraspinal VA was calculated as VA = (1 − sTw/eRTw) × 100% (27). In compliance with the recent Todd et al. (28) recommendations to ensure the quality of the TMS data, (1) VM MEP amplitude during contractions at 50–100% MVC was >40% Mmax; (2) BF MEP amplitude was small, see Figure 1C for exemplar data; (3) estimated resting twitches derived from linear regressions with correlation coefficients of <0.8 were excluded, and on the basis of this criterion, supraspinal VA data are presented for only seven of nine participants; and (4) *r*-values for the linear regression performed to determine the estimated resting twitch were 0.92 ± 0.02 (placebo) and 0.91 ± 0.02 (caffeine) for the seven participants included.

### Statistical Analyses

Data are presented as mean ± SEM. Performance and neuromuscular function data were statistically analyzed by two-way repeated measures ANOVA (condition: placebo vs caffeine; time: parameter-dependent number of levels). Blood and muscle metabolite parameters were analyzed by three-way repeated measures ANOVA for main and interaction effects of condition: placebo vs caffeine; exercise set: one to five levels, and time (first 110-s exercise and Tlim, muscle data; pre- vs post-sets; blood data). The significant main effects were followed up with *t*-tests with Bonferroni correction for multiple comparisons, and significant interaction effects were followed up with *post hoc* two-way ANOVA, as appropriate. The overall acceptable significance level of differences for all statistical tests was set at *p* < 0.05. The statistical analyses were performed in SPSS version 23 (SPSS Inc., Chicago, IL, USA).

## Results

### Effect of Caffeine Ingestion in Resting Muscle

Mmax amplitude and area in the resting muscle were not significantly altered 1 h after placebo or caffeine ingestion. There was a tendency for MEP amplitude to increase after caffeine ingestion (*p* = 0.07, η^2^ = 0.353, main condition effect, Table 1) but the caffeine effect on MEP area (84.7 ± 48.2 vs 14.1 ± 14.5%, *p* = 0.216) did not reach statistical significance. Peak twitch amplitude elicited by PNS in the resting muscle was not affected by placebo or caffeine ingestion; however, twitch contraction time (CT) was significantly shorter in the caffeine trial (−16.0 ± 5.2%, *p* = 0.028, main condition effect, Table 1).

**Table 1:** Parameters (mean ± SEM, *n* = 9) of the cortical (motor-evoked potentials, MEPs) and peripheral electrical (compound motor action potentials, Mmax) and mechanical (twitch, Tw) responses evoked by peripheral electrical nerve (PNS) and transcranial magnetic (TMS) stimulation in resting *musculus vastus medialis* before and 1 h after ingestion of placebo or caffeine supplements.

Parameters	Placebo		Caffeine	
	Pre-pill	1 h post pill	Pre-pill	1 h post pill
**I. PNS**				
Mmax amplitude (mV)	5.1 ± 0.7	4.6 ± 0.6	4.3 ± 0.5	3.4 ± 0.6
Mmax area (mV·ms)	33.2 ± 5.0	29.0 ± 5.5	26.6 ± 3.5	22.1 ± 4.3
Resting Tw amplitude (N)	17.3 ± 2.0	16.9 ± 2.0	16.0 ± 1.5	15.0 ± 1.2
Contraction time (ms)^a^	48.2 ± 6.0	54.5 ± 5.5	44.2 ± 3.3	42.5 ± 2.5

**II. TMS**				
MEP amplitude (% Mmax amplitude)	10.1 ± 2.2	8.5 ± 1.3	11.2 ± 2.3	17.9 ± 6.1
MEP area (% Mmax area)	14.6 ± 3.9	12.4 ± 2.1	13.7 ± 2.5	27.8 ± 10.8

*^a^Main condition effect*.

### Exercise Performance

**Figure 2:**
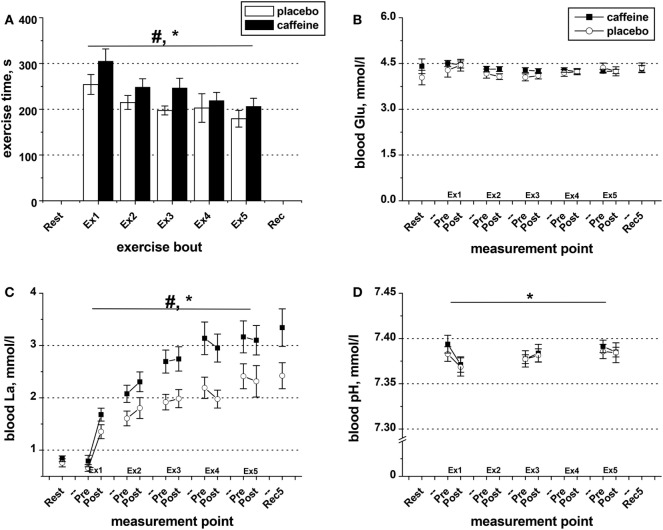
Exercise duration and blood metabolite data. **(A)** Population average (±SEM, *n* = 9) time of exercise to task failure throughout the five sets (Ex1–Ex5) completed by the participants 1 h after taking caffeine (filled bars) or placebo (open bars) supplements. Changes in blood pH **(D)** as well as in the concentrations of glucose **(B)** and lactate **(C)** in the blood samples collected before and after each exercise set in the trials with caffeine (black squares) and placebo (open circles); ANOVA, *p* < 0.05: ^#^main condition effect; *main time effect; ^†^set × time interaction effect.

Exercise time-to-task failure was significantly longer in the caffeine trial (*p* = 0.016, main condition effect), with the total exercise time accrued over the five exercise sets of 17.9 ± 6.0% longer in the caffeine (1,225 ± 86 s) than in the placebo trial (1,049 ± 73 s, *p* = 0.016). The time-to-task failure was decreased by 27.1 ± 7.8% and 31.2 ± 5.5% from sets 1 to 5 in the placebo and caffeine trials, respectively (*p* = 0.011, main time effect, Figure 2A). The difference between conditions remained consistent across the exercise sets. Similarly, the extent of the reduction in the maximum isometric contraction force at task failure after the five sets of knee extensor exercise (*p* = 0.003; main time effect) was not different between conditions (−12.9 ± 2.9%, placebo; −11.4 ± 5.8%, caffeine, Table 2). EMG RMS amplitude during MVC decreased (*p* = 0.008, main time effect) and MDF increased (*p* = 0.038, main time effect) similarly after the exercise sets in both conditions (Table 2).

**Table 2:** Parameters (mean ± SEM, *n* = 9 for all parameters except eRTw and sTw TMS where *n* = 7) of the *musculus vastus medialis* (VM) function evaluated with electrical peripheral nerve (PNS) and transcranial magnetic (TMS) stimulation and before (PreEx) and after (PostEx) repeated knee extensor exercise to fatigue performed 1 h after ingestion of placebo or caffeine supplements.

Parameters	Stimulus	Placebo		Caffeine	
		PreEx	PostEx	PreEx	PostEx
**I. Resting muscle**					
potTw amplitude (% MVC)^a^	PNS	24.7 ± 1.8	20.4 ± 2.1	24.4 ± 1.3	18.5 ± 1.5
eRTw (%MVC)^a^	TMS	21.2 ± 6.5	17.6 ± 4.6	22.5 ± 5.0	16.1 ± 3.8

**II. Knee extensors performing maximal voluntary isometric contraction (MVC)**					
Force (N)^a^		433.7 ± 25.6	376.0 ± 21.6	453.6 ± 12.5	402.0 ± 20.5
VM EMG MDF (Hz)^a^		81.3 ± 6.8	92.4 ± 9.1	87.5 ± 8.0	94.2 ± 7.4
VM EMG RMS amplitude (mV)^a^		0.22 ± 0.03	0.18 ± 0.01	0.21 ± 0.03	0.15 ± 0.02
VM EMG RMS amplitude (%Mmax)^c^		4.04 ± 0.71	4.68 ± 1.09	4.84 ± 0.64	3.82 ± 0.72
sTw amplitude (% MVC)^b^	PNS	3.7 ± 1.1	3.7 ± 0.9	2.5 ± 0.5	2.1 ± 0.5
sTw amplitude (% MVC)	TMS	2.3 ± 0.6	2.0 ± 0.7	1.2 ± 0.3	1.7 ± 0.4
CT (ms)^b^	PNS	29.3 ± 3.8	29.9 ± 3.4	23.6 ± 2.3	24.9 ± 2.9
CT (ms)	TMS	26.5 ± 3.2	21.4 ± 0.4	17.3 ± 2.3	18.3 ± 2.7
HRT (ms)	PNS	17.5 ± 0.8	17.4 ± 1.9	17.0 ± 1.1	15.3 ± 1.7
RR (1 s^−1^)^a,b^	TMS	−11.4 ± 1.0	−12.1 ± 1.3	−12.5 ± 0.9	−14.9 ± 0.9
VM CSP (ms)	TMS	201.5 ± 17.3	201.3 ± 15.4	210.3 ± 16.8	209.5 ± 22.1

*potTw, potentiated twitch; sTw, twitch superimposed during MVC; eRTw, estimated resting twitch; MDF, median frequency of the EMG power spectral density; Mmax, maximal M-wave amplitude; CT, twitch contraction time; HRT, twitch half relaxation time; RR, twitch relaxation rate; CSP, cortical silent period*.

*^a^Main time effect*.

*^b^Main condition effect*.

*^c^Interaction effect*.

### Blood Metabolite Data

Blood glucose concentration was not affected by exercise or by condition (Figure 2B). However, blood lactate concentration increased in response to exercise (*p* = 0.001, main time effect, Figure 2C), and the levels were significantly higher in the caffeine condition (*p* = 0.006, condition effect). The decrease in blood pH observed after the first bout of exercise in both conditions remained throughout the protocol (*p* = 0.039, main time effect, Figure 2D) and was not different between conditions.

### Muscle Metabolite Data

**Figure 3:**
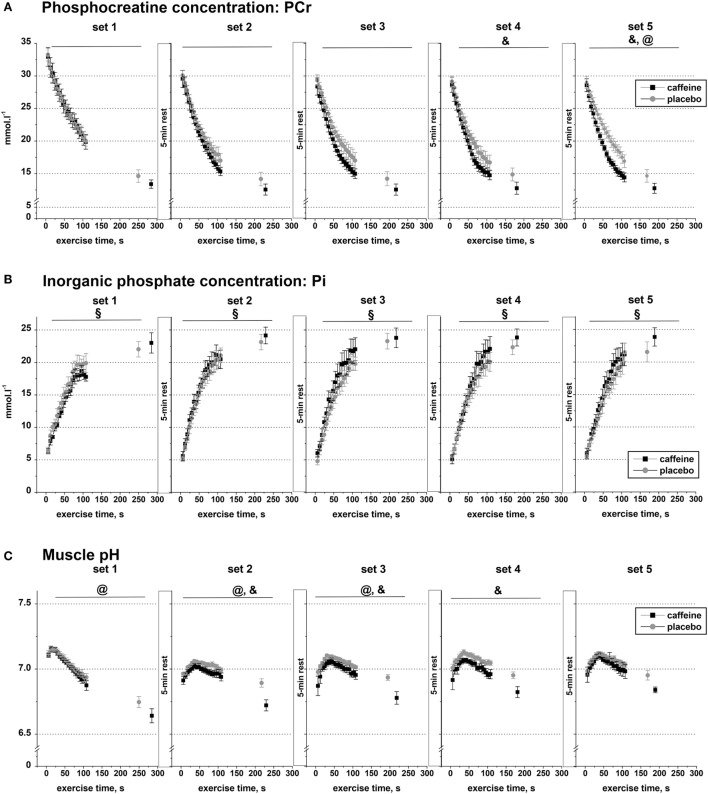
Muscle metabolite data. Population average (±SEM, *n* = 9) timeline of the changes in the concentrations of phosphocreatine **(A)**, inorganic phosphate **(B)**, and muscle pH **(C)** throughout the five sets of exercise (sets 1–5) completed to task failure by the participants 1 h after taking caffeine (black squares) or placebo (open circles) supplements. ^31^P-MRS spectra were calculated for every 6.0 s and are shown continuously up to 110 s of exercise together with their levels at the respective times of exhaustion; *post hoc p* < 0.05: ^&^condition effect; ^§^time effect; ^@^condition by time interaction effect.

Muscle PCr declined significantly during exercise (main effect of time, *p* < 0.001) and across sets (main effect of set, *p* < 0.001), and there were significant interaction effects for condition by set (*p* = 0.024), set by time (*p* < 0.001), and condition by set by time (*p* = 0.002; Figure 3A). *Post hoc* analysis revealed that muscle PCr concentration was significantly lower in the caffeine condition during sets four to five (*p* < 0.05). In addition, the PCr decline during the first 110 s of exercise and at Tlim was significantly greater in the caffeine than in the placebo condition during set five (*p* < 0.05, condition by time interaction).

Inorganic phosphate concentration increased significantly in each of the five sets (*p* < 0.001), but there was no significant condition effect or condition by time interaction effect in any of the five exercise sets (Figure 3B).

Muscle pH decreased significantly during exercise (main effect of time, *p* < 0.001) and across sets (main effect of set, *p* = 0.034), and there were significant interaction effects for condition by set (*p* = 0.01), condition by time (*p* < 0.001), and set by time (*p* < 0.001, Figure 3C). *Post hoc* analysis revealed that pH was significantly lower in the caffeine condition during sets two to four (*p* < 0.05). In addition, the pH decline during the first 110 s of exercise and at Tlim was significantly greater in the caffeine than in the placebo condition during sets 1–3 (*p* < 0.05, condition by time interaction effects).

### Effect of Caffeine on Peripheral Contractility before and after Exercise to Task Failure

#### Twitch Force in Relaxed Muscle

The amplitudes of the potentiated twitch (potTw) normalized to MVC (*p* < 0.001, main effect of time) and the normalized estimated resting twitch evoked by PNS and TMS, respectively, were lower after the fatiguing exercise (*p* = 0.01, main effect of time), but there was no difference between conditions (Table 2).

#### Twitch Parameters during MVC

The normalized peak twitch elicited during an MVC by PNS was smaller (*p* = 0.024, main condition effect, Table 2) in the caffeine than in the placebo trial but was not significantly altered by exercise. Normalized peak twitch elicited during an MVC by TMS was not affected by exercise or caffeine (Table 2). The CTs of the twitch evoked with PNS (*p* = 0.009, main condition effect) were shortened and tended to be shortened with TMS (*p* = 0.091, η^2^ = 0.316, main condition effect) in the caffeine trial relative to those in the placebo trial. The twitch RR evaluated with TMS increased after fatiguing exercise in both conditions (*p* = 0.006, main time effect) but was significantly faster in the caffeine trial (*p* = 0.003, main condition effect). The HRT time of the twitch evoked with PNS was not significantly affected by caffeine or fatiguing exercise (Table 2).

### Effect of Caffeine on Peripheral Muscle Excitability before and after Exercise to Task Failure

**Figure 4:**
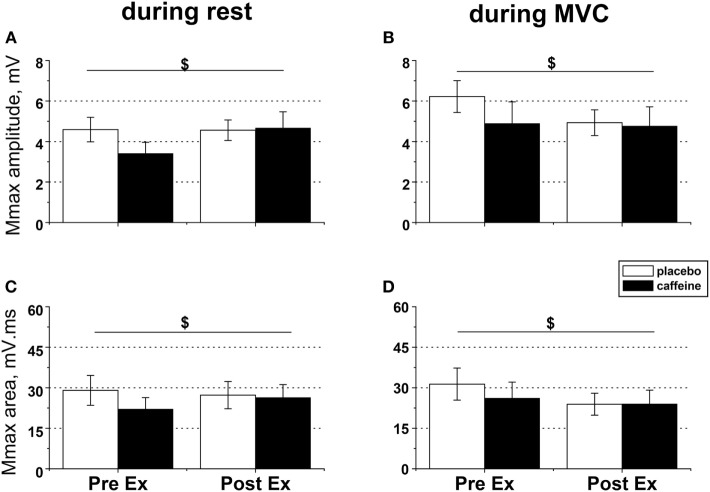
EMG responses to peripheral nerve stimulation in fresh and fatigued *musculus vastus medialis*. Population average (±SEM, *n* = 9) peak-to-peak amplitude **(A,B)** and total area **(C,D)** of the maximal compound muscle action potentials (Mmax) were calculated from the EMG responses evoked by suprathreshold (130% Mmax) femoral nerve stimulation. The femoral nerve was stimulated with five single pulses at rest **(A,C)** and during maximal voluntary contractions **(B,D)** performed before (PreEx) and after (PostEx) the completion of the exercise protocol to task failure. The exercise was conducted 1 h after ingestion of either caffeine (filled bars) or placebo (empty bars) supplement; ^$^condition × time interaction effect, *p* < 0.05.

In relaxed muscle, both Mmax amplitude (*p* = 0.03, condition × time interaction, Figure 4A) and area (*p* = 0.049; condition × time interaction, Figure 4C) increased after fatiguing exercise in the caffeine trial but decreased in the placebo trial. Caffeine prevented the reduction in M-wave amplitude and area during MVC that occurred after fatiguing exercise in the placebo trial (both *p* = 0.039, condition × time interaction effect, Figures 4B,D).

### Effect of Caffeine on Corticospinal Excitability before and after Exercise to Task Failure

**Figure 5:**
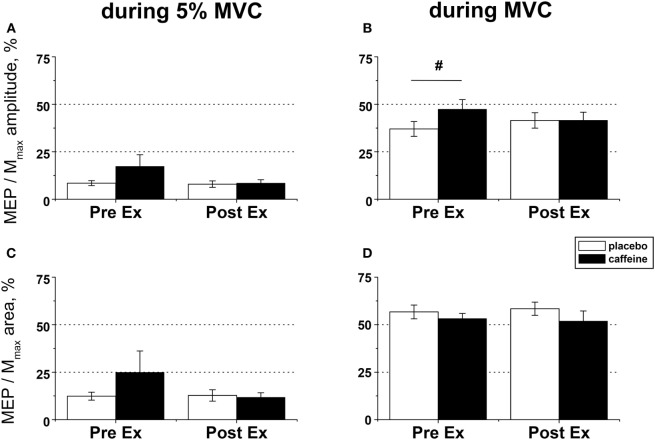
EMG responses to motor cortical stimulation in fresh and fatigued *musculus vastus medialis*. Population average (±SEM, *n* = 9) peak-to-peak amplitude **(A,B)** and total area **(C,D)** of the motor-evoked potentials (MEPs) were calculated from the EMG responses evoked by the transcranial magnetic stimulation (TMS) of the motor cortex area affiliated with the lower limb muscles and normalized to the respective parameters of the maximal compound muscle action potentials (Mmax) evoked during maximal voluntary contractions (MVCs) by suprathreshold (130% Mmax) femoral nerve stimulation. The motor cortex was stimulated during low (5% MVC) and maximal (100% MVC) voluntary contractions performed before (PreEx) and after (PostEx) the completion of the exercise protocol to task failure. The exercise was conducted 1 h after ingestion of either caffeine (filled bars) or placebo (empty bars) supplement. At each stimulation point, five single TMS pulses were delivered at suprathreshold intensity (120% of the active motor threshold identified at a muscular contraction of 5% MVC strength); *post hoc p* < 0.05: ^§^condition effect.

Motor-evoked potential amplitude and area evoked by TMS in the muscle maintaining a low-level contraction (~5% MVC) were not significantly affected by caffeine supplementation or fatiguing exercise (Figures 5A,C). By contrast, the MEP amplitude in the maximally contracting muscle was significantly higher pre-exercise in the caffeine than in the placebo condition (*post hoc* analysis, *p* = 0.007) and tended to increase by 17.0 ± 12.8% in the placebo trial but to decrease on average by −20.1 ± 8.4% in the caffeine trial after the fatiguing exercise (*p* = 0.09, η^2^ = 0.323, condition × time interaction) such that MEP amplitudes were similar in both conditions at volitional fatigue (Figure 5B). However, neither MEP area during MVC (Figure 5D) nor cortical silent period (Table 2) was affected significantly by caffeine or fatiguing exercise.

### Effect of Caffeine on VA before and after Exercise to Task Failure

**Figure 6:**
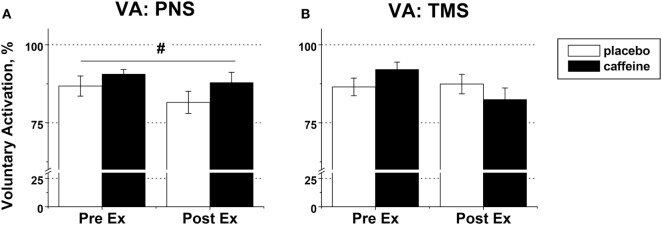
Voluntary activation (VA) in fresh and fatigued knee extensors. Population average (±SEM, %) values of the peripheral (*n* = 9) **(A)** and supraspinal (*n* = 7) **(B)** VA were calculated using the standard twitch interpolation equations from the twitch responses to peripheral femoral nerve (PNS) and transcranial magnetic (TMS) stimulation. Stimulation pulses were delivered during and 2 s after the maximal voluntary contractions performed before (PreEx) and after (PostEx) the exercise to task failure. The exercise was completed 1 h after ingestion of either caffeine (filled bars) or placebo (empty bars) supplement; *p* < 0.05: ^#^main condition effect.

Peripheral VA was significantly higher in the caffeine than in the placebo trial (*p* = 0.007, main condition effect, Figure 6A). Cortical VA also tended to be higher in the caffeine than in the placebo trials before exercise but declined to a greater extent during exercise (*p* = 0.074, η^2^ = 0.437, condition × time interaction effect, Figure 6B). EMG RMS amplitude normalized to Mmax amplitude was higher pre-exercise and lower at task failure in the caffeine than in the placebo trial (*p* = 0.033, condition × time interaction effect, Table 2).

## Discussion

Peripheral and central neural processes were both involved in the caffeine-induced extension of time-to-task failure (~18%) in each of the five high-intensity knee extensor exercise sets. Caffeine increased pre-exercise central excitability and motor drive as indicated by the elevated corticospinal excitability and peripheral VA, thus allowing participants to continue to exercise beyond the degree of metabolic perturbation achieved in the placebo trial, with significantly lower muscle (PCr) and pH evident at task failure in the caffeine trial. Corticospinal excitability and supraspinal VA declined to similar levels at task failure in both conditions, collectively implicating central mechanisms, possibly adenosine receptor antagonism. The extended exercise time in the caffeine trials resulted in greater peripheral metabolic perturbations (lower muscle PCr and pH) but these were accompanied with the better preservation of peripheral membrane excitability at task failure, most likely due to the previously observed reductions in interstitial potassium accumulation. In addition, muscle contractility was enhanced after caffeine ingestion with faster muscle contraction and RRs indicative of altered myofibrillar calcium kinetics and sensitivity. Similar levels of VA and corticospinal excitability were evident at task failure in both caffeine and placebo conditions, despite greater disturbance of the metabolic milieu. Collectively, the current data indicate that caffeine-induced increases in central motor drive and corticospinal excitability were attenuated at task failure. This may have been induced by the afferent feedback of the greater disturbance of the muscle milieu, resulting in a stronger inhibitory input to the spinal and supraspinal motor neurons. However, causality needs to be established through further experiments.

Caffeine has consistently been shown to improve the performance of short-term high-intensity exercise (2). There is a growing consensus that the ergogenic effects are centrally mediated through the antagonism of adenosine receptors (5). It is suggested that this causes increased central drive due to an increased excitatory neurotransmitter release and central excitatory input, thus lowering alpha-motoneuron activation threshold and increasing neural excitability (12, 13). In the present study, caffeine ingestion increased peripheral VA, as well as EMG RMS amplitude normalized to the maximal M-wave amplitude in fresh muscle, indicating increased central drive, most likely *via* optimized motor unit recruitment and firing rates. The elevation in MEP amplitude in fresh muscle maintaining either a brief maximal contraction or a very low-level contraction (5% MVC) provides evidence of the increased corticospinal excitability anticipated to result from adenosine receptor antagonism. At task failure, supraspinal VA and corticospinal excitability were similar for both conditions, although it took ~30% longer to reach this point in the caffeine trial. This suggests that the caffeine-induced increases in central excitability and drive are pivotal for the observed ergogenic effects. The extended exercise time and greater contractile work observed in the caffeine trial occurred despite greater metabolic perturbations. A modulatory influence is exerted on the spinal motoneurons and supraspinal drive by sensory feedback from group III and IV muscle afferents regarding changes in the muscle metabolites, including PCr depletion and accumulation of H^+^ and Pi (29). It seems that task failure occurred when caffeine-induced increases in central motor drive were canceled by the stronger inhibitory input *via* the muscle afferents to prevent perturbation of the muscle milieu beyond a “critical threshold” (30).

In contrast to corticospinal excitability (MEP amplitude), peripheral excitability (Mmax amplitude) was not affected by caffeine ingestion in fresh muscle, which supports the importance of the spinal and supraspinal processes to caffeine’s ergogenic effects. However, caffeine ingestion resulted in the better preservation of peripheral excitability in maximally contracting muscle and increased peripheral excitability in the relaxed muscle after task failure. The accumulation of extracellular potassium $\left(K_{o}^{+}\right)$ has been implicated in the loss of peripheral excitability during high-intensity exercise. *In vitro* studies find that twitch and tetanic force declines as K+ exceeds 8 mM and contractile force is eliminated at 12 mM K+ (31). *In vivo* studies support these findings with reports of muscle interstitial K+ concentrations of 10–12 mM at the point of exhaustion after intense exercise (21, 32, 33). This deterioration in function seems to be related to a progressive depolarization of the resting membrane potential (31) which would reduce M-wave amplitude (34). Unfortunately, neither venous nor interstitial (K+) was measured in the present study, but Mohr et al. (21) demonstrated that caffeine ingestion both improved exercise performance and attenuated the increase in interstitial potassium during single-leg knee extensor exercise, similar to that employed in the present study. The preservation of peripheral excitability in the caffeine condition may therefore be attributable to reduced $K_{o}^{+}$ accumulation.

In addition, muscle pH was significantly lower in the caffeine condition in the present study, and low pH may reinforce the potassium efflux *via* the opening of pH-sensitive K+-channels (33, 35) and therefore contribute to peripheral fatigue caused by interstitial K+ accumulation. However, despite this, peripheral excitability was higher in the caffeine condition at task failure. Interestingly, *in vitro* acidosis has been demonstrated to preserve T-tubule excitability through lower Cl conductance, which reduces the magnitude of Na+ current required to propagate an action potential (36). Using a preparation from rat extensor digitorum longus (EDL) muscle, Hansen et al. (37) demonstrated that lactic acid and catecholamines are each able to counteract the detrimental effect of $K_{o}^{+}$ accumulation on membrane excitability. The former through lower Cl^−^ conductance and the latter *via* enhanced Na(+)−K+ pump activity *via* adenylate cyclase signaling. However, these mechanisms have not yet been confirmed in the human *in vivo* setting and will be offset by the well-documented detrimental effects of low pH on enzyme activity and metabolism.

The temporal parameters of the twitch evoked by PNS or TMS in relaxed muscle and during maximal contractions were the main measures employed in the present study to evaluate the effect of caffeine on fatigue-induced changes in muscle contractility during exercise. CT was consistently shorter after caffeine ingestion both in fresh and in fatigued muscle, indicating faster Ca^2+^ release or greater Ca^2+^ sensitivity. Tarnopolsky and Cupido (4) also found that contractility was increased after caffeine ingestion. Specifically, the twitch amplitude induced by low-frequency stimulation trains delivered to the common peroneal nerve during dorsiflexion was increased after caffeine ingestion in healthy male participants, which was attributed to increased Ca^2+^ release from the SR. Tallis et al. (18) found that twitch force in isolated mouse soleus and EDL was elevated by 6% at physiologically relevant levels as 50–70 μM (caffeine) with effects observed during shortening, thus implicating Ca^2+^ release. In the present study, RR, which is suggested to be indicative of Ca^2+^ reuptake into the SR, was accelerated by caffeine and was also faster in the fatigued muscle in both conditions. Most likely, this is due to increased muscle temperature, which has been shown to increase RR (38) and counteract the fatigue-induced slowing of the muscle RR. Although not measured in the present study, muscle temperature is anticipated to be higher in the caffeine condition, due to 30% longer exercise duration in the caffeine condition, and we cannot eliminate the influence of this factor on the observed neuromuscular effects of caffeine.

In conclusion, caffeine supplementation extended the time-to-task failure during high-intensity knee extensor exercise despite greater changes in muscle metabolites, due to modulation of the interplay between central and peripheral neural processes. Central motor drive and corticospinal excitability were enhanced after caffeine ingestion, which allowed exercise to continue beyond the degree of muscle acidosis and PCr depletion achieved in the placebo trial. Caffeine preserved peripheral excitability at task failure probably due to lesser $K_{o}^{+}$ accumulation and lower pH, allowing the maintenance of the generation and conduction of the membrane potentials. Contractility was also enhanced by caffeine supplementation in both fresh and fatigued muscle, most likely due to faster Ca^2+^ release. Caffeine is a potent stimulant that extends time-to-task failure and hence allows greater disturbance of the muscle milieu to occur before task failure, thus improving exercise performance.

## Ethics Statement

Nine male recreational athletes [age: 26.0 ± 2.7 (±SEM) years; height: 1.79 ± 0.02 m; weight: 78.4 ± 2.2 kg] took part in this double-blind randomized controlled study, which was approved by the Sport and Health Sciences Research Ethics Committee at the Exeter University and conducted in accordance with the Declaration of Helsinki. After being informed verbally and in writing of the experimental procedures and associated risks, all participants completed a medical health questionnaire and a screening questionnaire to ensure that there were no contraindications for magnetic resonance scanning and cortical stimulation, before providing written informed consent to the experimental procedures.

## Author Contributions

All authors contributed to the concept and design of the study, acquisition, analysis and interpretation of the data, drafting and final approval of the manuscript, and all authors agree to be accountable for the content of the work.

## Conflict of Interest Statement

The authors declare that the research was conducted in the absence of any commercial or financial relationships that could be construed as a potential conflict of interest.

## References

[1] Warren GL Park ND Maresca RD Mckibans KI Millard-Stafford ML. Effect of caffeine ingestion on muscular strength and endurance: a meta-analysis. Med Sci Sports Exerc (2010) 42:1375–87. 10.1249/MSS.0b013e3181cabbd8 20019636

[2] Astorino TA Roberson DW. Efficacy of acute caffeine ingestion for short-term high-intensity exercise performance: a systematic review. J Strength Cond Res (2010) 24:257–65. 10.1519/JSC.0b013e3181c1f88a 19924012

[3] Woolf K Bidwell WK Carlson AG. The effect of caffeine as an ergogenic aid in anaerobic exercise. Int J Sport Nutr Exerc Metab (2008) 18:412–29. 10.1123/ijsnem.18.4.412 18708685

[4] Tarnopolsky M Cupido C. Caffeine potentiates low frequency skeletal muscle force in habitual and nonhabitual caffeine consumers. J Appl Physiol (2000) 89:1719–24. 10.1152/jappl.2000.89.5.1719 11053318

[5] Davis JM Zhao ZW Stock HS Mehl KA Buggy J Hand GA. Central nervous system effects of caffeine and adenosine on fatigue. Am J Physiol Regul Integr Comp Physiol (2003) 284:R399–404. 10.1152/ajpregu.00386.2002 12399249

[6] Allen DG Westerblad H The effects of caffeine on intracellular calcium, force and the rate of relaxation of mouse skeletal muscle. J Physiol (1995) 487:331–42. 10.1113/jphysiol.1995.sp020883 8558467 PMC1156576

[7] Lindinger MI Graham TE Spriet LL Caffeine attenuates the exercise induced increase in plasma [K+] in humans. J Appl Physiol (1993) 74:1149–55. 10.1152/jappl.1993.74.3.1149 8387071

[8] Fredholm BB Chen JF Masino SA Vaugeois JM. Actions of adenosine at its receptors in the CNS: insights from knockouts and drugs. Annu Rev Pharmacol Toxicol (2005) 45:385–412. 10.1146/annurev.pharmtox.45.120403.095731 15822182

[9] Elmenhorst D Meyer PT Matusch A Winz OH Bauer A Caffeine occupancy of human cerebral A(1) adenosine receptors: *in vivo* quantification with F-18-CPFPX and PET. J Nucl Med (2012) 53:1723–9. 10.2967/jnumed.112.105114 22966134

[10] Phillis JW Edstrom JP Effects of adenosine-analogs on rat cerebral cortical-neurons. Life Sci (1976) 19:1041–53. 10.1016/0024-3205(76)90296-4 994710

[11] Berkowitz BA Spector S Effect of caffeine and theophylline on disposition of brain serotonin in rat. Eur J Pharmacol (1971) 16:322 10.1016/0014-2999(71)90034-3 5132559

[12] Barasi S Roberts MHT Effects of stimulation of nucleus raphe and iontophoretically applied 5-hydroxytryptamine on spinal motoneurons. J Physiol (1974) 236:P11–2. 4818485

[13] Walton C Kalmar JM Cafarelli E. Effect of caffeine on self-sustained firing in human motor units. J Physiol (2002) 545:671–9. 10.1113/jphysiol.2002.025064 12456842 PMC2290683

[14] Kalmar JM Cafarelli E Central excitability does not limit postfatigue voluntary activation of quadriceps femoris. J Appl Physiol (2006) 100:1757–64. 10.1152/japplphysiol.01347.2005 16424071

[15] Del Coso J Estevez E Mora-Rodriguez R. Caffeine during exercise in the heat: thermoregulation and fluid-electrolyte balance. Med Sci Sports Exerc (2009) 41:164–73. 10.1249/MSS.0b013e318184f45e 19092693

[16] Allen DG Lamb GD Westerblad H. Skeletal muscle fatigue: cellular mechanisms. Physiol Rev (2008) 88:287–332. 10.1152/physrev.00015.2007 18195089

[17] James RS Kohlsdorf T Cox VM Navas CA 70 μM caffeine treatment enhances *in vitro* force and power output during cyclic activities in mouse extensor digitorum longus muscle. Eur J Appl Physiol (2005) 95:74–82. 10.1007/s00421-005-1396-2 15959797

[18] Tallis J James RS Cox VM Duncan MJ. The effect of physiological concentrations of caffeine on the power output of maximally and submaximally stimulated mouse EDL (fast) and soleus (slow) muscle. J Appl Physiol (2012) 112:64–71. 10.1152/japplphysiol.00801.2011 21979804

[19] Fortune E Lowery MM. Effect of extracellular potassium accumulation on muscle fiber conduction velocity: a simulation study. Ann Biomed Eng (2009) 37:2105–17. 10.1007/s10439-009-9756-4 19588250

[20] Shushakov V Stubbe C Peuckert A Endeward V Maassen N. The relationships between plasma potassium, muscle excitability and fatigue during voluntary exercise in humans. Exp Physiol (2007) 92:705–15. 10.1113/expphysiol.2006.036384 17434915

[21] Mohr M Nielsen JJ Bangsbo J. Caffeine intake improves intense intermittent exercise performance and reduces muscle interstitial potassium accumulation. J Appl Physiol (2011) 111:1372–9. 10.1152/japplphysiol.01028.2010 21836046

[22] Bailey SJ Fulford J Vanhatalo A Winyard PG Blackwell JR DiMenna FJ Dietary nitrate supplementation enhances muscle contractile efficiency during knee-extensor exercise in humans. J Appl Physiol (2010) 109:135–48. 10.1152/japplphysiol.00046.2010 20466802

[23] Stevens-Lapsley JE Thomas AC Hedgecock JB Kluger BM. Corticospinal and intracortical excitability of the quadriceps in active older and younger healthy adults. Arch Gerontol Geriatr (2013) 56:279–84. 10.1016/j.archger.2012.06.017 22951029 PMC3502668

[24] Rainoldi A Melchiorri G Caruso I. A method for positioning electrodes during surface EMG recordings in lower limb muscles. J Neurosci Methods (2004) 134:37–43. 10.1016/j.jneumeth.2003.10.014 15102501

[25] Taylor DJ Styles P Matthews PM Arnold DA Gadian DG Bore P Energetics of human muscle: exercise induced ATP depletion. Magn Reson Med (1986) 3:44–54. 10.1002/mrm.1910030107 3959889

[26] Taylor DJ Bore PJ Styles P Gadian DG Radda GK. Bioenergetics of intact human muscle. A 31P nuclear magnetic resonance study. Mol Biol Med (1983) 1:77–94. 6679873

[27] Todd G Taylor JL Gandevia SC. Measurement of voluntary activation of fresh and fatigued human muscles using transcranial magnetic stimulation. J Physiol (2003) 551:661–71. 10.1113/jphysiol.2003.044099 12909682 PMC2343213

[28] Todd G Taylor JL Gandevia SC. Measurement of voluntary activation based on transcranial magnetic stimulation over the motor cortex. J Appl Physiol (2016) 121:678–86. 10.1152/japplphysiol.00293.2016 27418687

[29] Gandevia SC. Spinal and supraspinal factors in human muscle fatigue. Physiol Rev (2001) 81:1725–89. 10.1152/physrev.2001.81.4.1725 11581501

[30] Amann M Blain GM Proctor LT Sebranek JJ Pegelow DF Dempsey JA. Implications of group III and IV muscle afferents for high-intensity endurance exercise performance in humans. J Physiol (2011) 589:5299–309. 10.1113/jphysiol.2011.213769 21878520 PMC3225681

[31] Cairns SP Hing WA Slack JR Mills RG Loiselle DS Different effects of raised [K+](0) on membrane potential and contraction in mouse fast- and slow-twitch muscle. Am J Physiol Cell Physiol (1997) 273:C598–611. 10.1152/ajpcell.1997.273.2.C598 9277357

[32] Nordsborg N Mohr M Pedersen LD Nielsen JJ Langberg H Bangsbo J. Muscle interstitial potassium kinetics during intense exhaustive exercise: effect of previous arm exercise. Am J Physiol Regul Integr Comp Physiol (2003) 285:R143–8. 10.1152/ajpregu.00029.2003 12663256

[33] Mohr M Nordsborg N Nielsen JJ Pedersen LD Fischer C Krustrup P Potassium kinetics in human muscle interstitium during repeated intense exercise in relation to fatigue. Pflugers Arch (2004) 448:452–6. 10.1007/s00424-004-1257-6 15048574

[34] Sejersted OM Sjogaard G. Dynamics and consequences of potassium shifts in skeletal muscle and heart during exercise. Physiol Rev (2000) 80:1411–81. 10.1152/physrev.2000.80.4.1411 11015618

[35] Street D Nielsen JJ Bangsbo J Juel C. Metabolic alkalosis reduces exercise-induced acidosis and potassium accumulation in human skeletal muscle interstitium. J Physiol (2005) 566:481–9. 10.1113/jphysiol.2005.086801 15860529 PMC1464741

[36] Pedersen TH Nielsen OB Lamb GD Stephenson DG. Intracellular acidosis enhances the excitability of working muscle. Science (2004) 305:1144–7. 10.1126/science.1101141 15326352

[37] Hansen AK Clausen T Nielsen OB. Effects of lactic acid and catecholamines on contractility in fast-twitch muscles exposed to hyperkalemia. Am J Physiol Cell Physiol (2005) 289:C104–12. 10.1152/ajpcell.00600.2004 15743886

[38] Todd G Butler JE Taylor JL Gandevia SC. Hyperthermia: a failure of the motor cortex and the muscle. J Physiol (2005) 563:621–31. 10.1113/jphysiol.2004.077115 15613373 PMC1665582

